# Double-Layered Microphysiological System Made of Polyethylene Terephthalate with Trans-Epithelial Electrical Resistance Measurement Function for Uniform Detection Sensitivity

**DOI:** 10.3390/bios15100663

**Published:** 2025-10-02

**Authors:** Naokata Kutsuzawa, Hiroko Nakamura, Laner Chen, Ryota Fujioka, Shuntaro Mori, Noriyuki Nakatani, Takahiro Yoshioka, Hiroshi Kimura

**Affiliations:** 1 Micro/Nano Technology Center, Tokai University, Hiratsuka 259-1292, Kanagawa, Japan; 2 Department of Medicine, Division of Pulmonary Medicine, Tokai University School of Medicine, Isehara 259-1143, Kanagawa, Japan; 3 The Institute of Medical Sciences, Tokai University School of Medicine, Isehara 259-1143, Kanagawa, Japan; 4 SCREEN Holdings Co., Ltd., Kyoto 612-8486, Japan; 5 TOKYO OHKA KOGYO Co., Ltd., Koza 253-0114, Kanagawa, Japan

**Keywords:** microphysiological system (MPS), trans-epithelial electro resistance (TEER), microfluidic device, gut-on-a-chip, Intestinal epithelial model, polyethylene terephthalate (PET)

## Abstract

Microphysiological systems (MPSs) have emerged as alternatives to animal testing in drug development, following the FDA Modernization Act 2.0. Double-layer channel-type MPS chips with porous membranes are widely used for modeling various organs, including the intestines, blood–brain barrier, renal tubules, and lungs. However, these chips faced challenges owing to optical interference caused by light scattering from the porous membrane, which hinders cell observation. Trans-epithelial electrical resistance (TEER) measurement offers a non-invasive method for assessing barrier integrity in these chips. However, existing electrode-integrated MPS chips for TEER measurement have non-uniform current densities, leading to compromised measurement accuracy. Additionally, chips made from polydimethylsiloxane have been associated with drug absorption issues. This study developed an electrode-integrated MPS chip for TEER measurement with a uniform current distribution and minimal drug absorption. Through a finite element method simulation, electrode patterns were optimized and incorporated into a polyethylene terephthalate (PET)-based chip. The device was fabricated by laminating PET films, porous membranes, and patterned gold electrodes. The chip’s performance was evaluated using a perfused Caco-2 intestinal model. TEER levels increased and peaked on day 5 when cells formed a monolayer, and then they decreased with the development of villi-like structures. Concurrently, capacitance increased, indicating microvilli formation. Exposure to staurosporine resulted in a dose-dependent reduction in TEER, which was validated by immunostaining, indicating a disruption of the tight junction. This study presents a TEER measurement MPS platform with a uniform current density and reduced drug absorption, thereby enhancing TEER measurement reliability. This system effectively monitors barrier integrity and drug responses, demonstrating its potential for non-animal drug-testing applications.

## 1. Introduction

Microphysiological systems (MPSs) have recently emerged as an essential technology in drug discovery, providing a viable alternative to animal testing [1]. The US Food and Drug Administration’s Modernization Act 2.0 underscores the importance of addressing ethical and scientific concerns associated with animal testing, as well as the necessity for swift and accurate drug evaluation methods [2,3]. MPSs enable the in vitro replication of organ and tissue functions, making them applicable to a wide range of organ models. The double-layer channel-type MPS chip, featuring a porous membrane, has garnered attention as a versatile and convenient platform for organ models like the intestinal tract, blood–brain barrier, kidneys, and lungs in the drug discovery process [4,5]. However, a key challenge lies in evaluating cell conditions and kinetics for double-layer channel-type MPS chips with porous membranes. While the porous membrane facilitates compartmental separation, it also leads to diffuse light reflection, complicating the observation of cellular conditions through optical techniques. To address this issue, an evaluation method independent of optical means is imperative. To monitor cells on porous membranes, trans-epithelial electrical resistance (TEER) emerges as a promising parameter for a non-destructive and quantitative evaluation of cell barrier function.

TEER measurements, widely utilized in cell culture systems with culture inserts, are crucial for accurately assessing the state of cells on porous membranes [6,7,8]. Several studies have proposed integrating electrodes into double-layer channel-type MPS chips to measure TEER [9,10,11,12,13,14]. However, TEER measurements on MPS chips are still limited, and several problems persist with the existing methods. One issue is that the electrode geometry utilized for TEER measurement is not optimized for the cell culture area, leading to non-uniform current density during measurement, which can impact accuracy [14,15]. Additionally, differences in chip and electrode designs make it difficult to compare results across studies. For instance, double-layer channel-type MPS chips can perfuse media to culture cells with TEER measurement function, but their electrode designs and geometry differ [16]. Moreover, achieving a uniform cell monolayer in actual cell culture is challenging owing to potential issues like aggregates and detachment. Therefore, ensuring uniform sensitivity across the entire cell monolayer area is essential. To address these issues, Kamei et al. developed an electrode pattern that enhanced current density uniformity in the cell culture area within MPS chips through computer simulations using the finite element method (FEM) [17]. However, no study to date has experimentally validated a PET-based double-layer chip with optimized electrode geometry for uniform TEER measurement. Therefore, the development of a highly efficient MPS chip with TEER measurement capabilities is essential and imperative, and its effectiveness should be validated. Furthermore, existing MPS chips for TEER measurement are typically made of polydimethylsiloxane (PDMS), which can lead to drug adsorption and sorption issues that compromise the reliability of drug testing [18]. For instance, a study that evaluated differences in adsorption and absorption based on chip material found that a double-layer channel-type MPS chip made of PDMS showed adsorption of Rhodamine B, a hydrophobic small molecular dye, around the flow channel. Additionally, small molecular drugs, such as Nifedipine, Caumarin, and Bay K8644, were also adsorbed, demonstrating the importance of using non-adsorptive chip materials to evaluate drug discovery and development [19].

In this study, we aimed to develop a double-layer channel-type MPS chip with a uniform electrode pattern of current density designed using FEM simulation. We chose polyethylene terephthalate (PET) as the material for this project, owing to its low drug adsorption and sorption properties, to enhance the reliability of drug testing. Furthermore, we developed a technology to laminate and integrate PET films, porous membranes, and flow channel layers, successfully implementing an electrode pattern validated through FEM simulations on a PET-based chip. The MPS chip’s performance was then demonstrated using intestinal epithelial cells (Caco-2) as a model of the intestinal tract. By utilizing this model for drug testing, we accurately assessed cellular conditions, showcasing the potential of this device as a drug discovery platform with the capacity to replace animal testing. These findings offer valuable insights for optimizing the design and functionality of MPS chips, marking a significant advancement in their role as an alternative to animal testing in the drug discovery process.

## 2. Materials and Methods

### 2.1. Double-Layered TEER Measurement Microfluidic Chip

**Figure 1:**
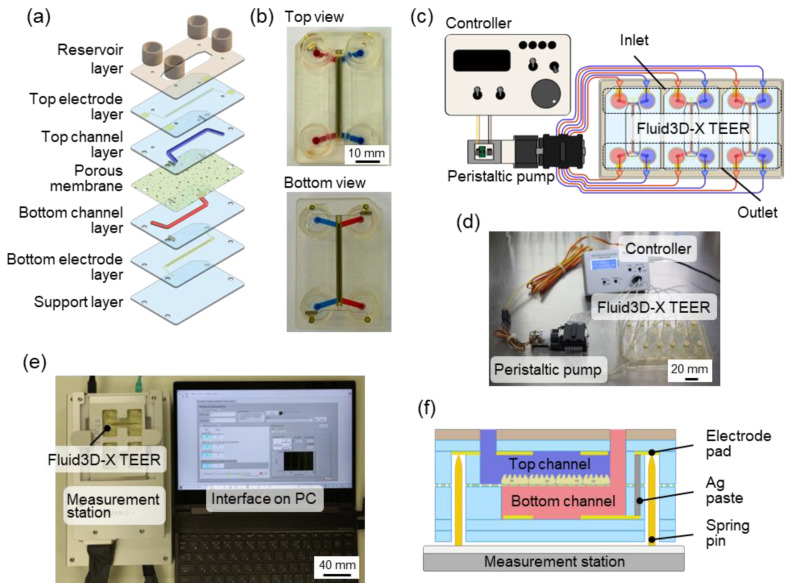
Fluid3D-X TEER and experimental setups of perfusion culture and TEER measurement. (**a**) Assembled view of Fluid3D-X TEER; the chip is fabricated by PET layer bonding. (**b**) Top and bottom images of Fluid3D-X TEER; reservoirs are installed in the microfluidic ports on the top layer. (**c**) Peristaltic pump setup for medium perfusion. (**d**) Actual setup of perfusion culture system using Fluid3D-X TEER. (**e**) Photograph of the TEER measurement system. Two chips are set in the measurement station. (**f**) Schematic of the contact between the electrode pad on the chip and the pin in the measurement station.

The TEER measurement microfluidic chip for perfusion cell culture utilized Fluid3D-X^®^, a product from Tokyo Ohka Kogyo in Kanagawa, Japan [20,21]. This innovative chip features a double-layered microchannel structure separated by a porous membrane, with medium chambers in each port [21,22]. The assembly of Fluid3D-X with the TEER measurement electrode (Fluid3D-X TEER) involved laminating PET films with microchannel structures, micropatterned Au electrodes, and a porous membrane (2000M12/640N453/A4, PET, pore size: 0.45 µm, it4ip, Louvain-laNeuve, Belgium) (Figure 1a,b). To perfuse the culture medium, the medium reservoirs on the three Fluid3D-X TEERs in an ANSI/SLAS-compliant rectangular plate were connected to a peristaltic pump (AQ-RP6R-001, Takasago Fluidic Systems, Nagoya, Japan) with silicone tubes (Figure 1c,d). A peristaltic pump controller arbitrarily controls the flow rate of the perfusion medium.

### 2.2. Electrode Design Through FEM Simulations

**Figure 2:**
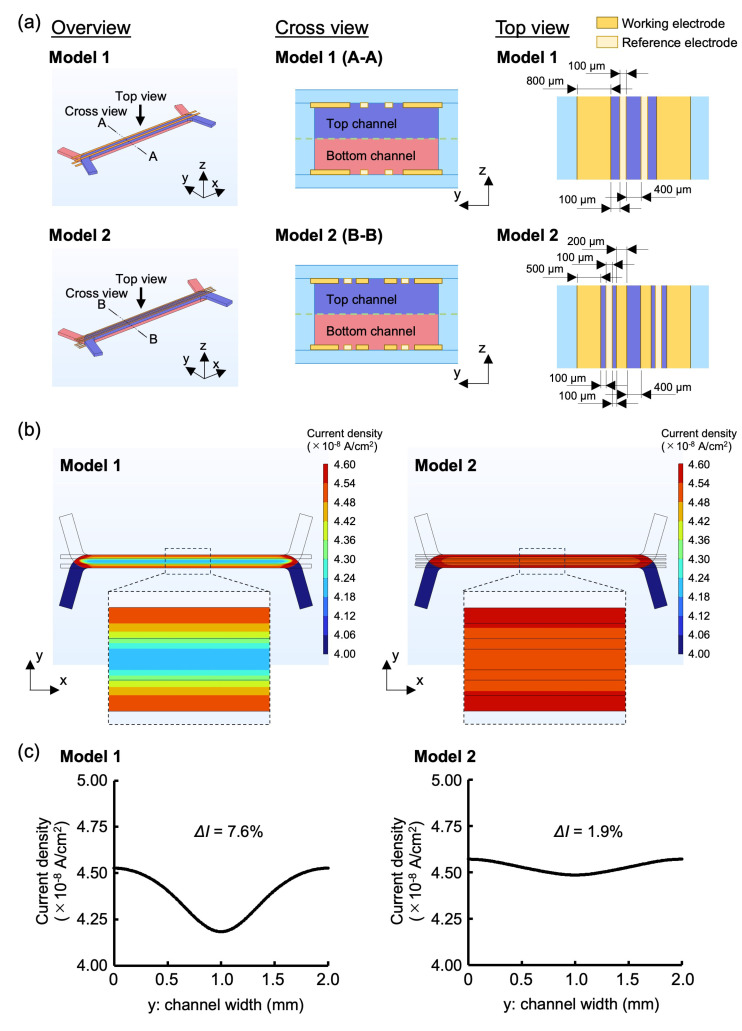
Optimization of Au electrode design on Fluid3D-X TEER via FEM simulation. (**a**) Microchannel and electrode pattern geometries for Model 1 and Model 2 (overview, cross view, and top view). (**b**) FEM simulation contour maps of current density near the porous membrane for Model 1 and Model 2. (**c**) Differences in current density distribution across the width of the microfluidic channels in the cell culture area between Model 1 and Model 2.

The accuracy and uniformity of cell measurements are significantly influenced by electrode placement and geometry [14]. To accurately assess the cell condition, the uniformity of the current applied to the porous membrane surface was assessed and utilized as the cell culture surface. Achieving uniformity of the applied current requires the strategic placement of electrodes across the entire surface relative to the measurement targets. While Au electrodes offer excellent biocompatibility, stability, and electrical properties, placing electrodes across the entire surface without obstructing cell observation can be challenging. To achieve highly accurate electrical measurements while maintaining cell observability, the current densities of the two-electrode geometries (Models 1 and 2) were simulated. COMSOL Multiphysics electrochemical modules were employed to assess the current density distribution using the FEM. Models 1 and 2 had an opening (400 μm wide) in the center of the channel for cell observation (Figure 2a). Only symmetrical electrode patterns along the central axis were considered in these models, as asymmetrical patterns in the longitudinal (y) direction of the cell culture areas are unsuitable for TEER measurements. Ensuring the uniformity of current passing through the cell surface is crucial for accurate TEER measurements. To evaluate the current density distribution in both the width (x) and longitudinal (y) directions of the channel at the cell surface, simulations were conducted. *ΔI* serves as an index of the variation in current density distribution within the evaluation range. A smaller *ΔI* indicates less variation in current density, signifying an ideal electrode shape.

(1) $$∆I\;\left[\%\right]=\frac{I_{max}-I_{min}}{I_{max}}\times 100$$

where *I_max_* and *I_min_* represent the maximum and minimum current densities, respectively. The specific parameters and boundary conditions utilized in this simulation are detailed in Tables S1 and S2. Notably, parameters other than the electrode design were the same for both models.

### 2.3. TEER Measurement Station

The TEER measurement system, comprising a laptop and measurement station, was developed as a dedicated measurement instrument for Fluid3D-X TEER. To measure the TEER, the Fluid3D-X TEERs were positioned on the measurement station (Figure 1e). The four-terminal method was employed to measure the potential difference between the upper and lower reference electrodes by applying a current to the working electrodes of both the upper and lower layers by contacting the spring pins of the TEER measurement station with the electrode pads of the Fluid3D-X TEER (Figure 1f). The four-terminal method offers advantages such as reduced susceptibility to resistance from the measurement circuit and wiring, ensuring high measurement stability even when the resistance of the measurement electrode section fluctuates owing to medium components. This system enabled the measurement of TEER (impedance) and capacitance. Further details regarding the system specifications can be found in Table S3.

### 2.4. Cell Culture Using Fluid3D-X TEER

Caco-2 cells were obtained from American Type Culture Collection (HTB-37, ATCC, Manassas, VA, USA) and maintained in DMEM (12320-032, Gibco, Thermo Fisher Scientific, Waltham, MA, USA) containing 10% Fetal Bovine Serum (10270106, Gibco, Thermo Fisher Scientific, Waltham, MA, USA), MEM Non-Essential Amino Acids solution (11140-050, Thermo Fisher Scientific, Waltham, MA, USA), and Penicillin-Streptomycin-Amphotericin B Suspension (161-23181, FUJIFILM Wako Pure Chemical Corporation, Osaka, Japan).

Prior to extracellular matrix coating, Fluid3D-X TEER was cooled to 4 °C. A 30 × diluted Matrigel^®^ Matrix Basement Membrane (356237, Corning, Corning, NY, USA) in FBS-free DMEM was then added to the top and bottom channels of Fluid3D-X TEER, followed by incubation at 4 °C overnight. Caco-2 cells cultured on a Petri dish, covering approximately 90%, were washed twice with phosphate-buffered saline (PBS) (14190144, Gibco Thermo Fisher Scientific, Waltham, MA, USA) and treated with trypsin (T4049, Sigma-Aldrich, St. Louis, MO, USA). Cells were suspended to 2 × 10^6^ cells/mL (2 × 10^5^ cells/cm^2^), and 110 μL of the cell suspension was seeded onto the top channel of Fluid3D-X TEER using a wide-pore tip micropipette. Subsequently, 120 µL of DMEM was injected into the bottom channel of Fluid3D-X TEER, and Fluid3D-X TEER in the plate was placed in a CO_2_ incubator (37 °C, 5% CO_2_) to allow for cell attachment to the porous membrane. After validating cell attachment within 4 to 5 h, a peristaltic pump was connected to the perfusion system, and 650 μL of DMEM was simultaneously added to the medium reservoirs of both the inlet and outlet using a multipipette from the top to the bottom channel. Following the initial filling of the medium in the reservoirs, medium perfusion was performed at a speed of 3.8 µL/min (pre-perfusion). On day 2, the medium was replaced, and the perfusion speed was increased to 15.2 µL/min. Subsequently, the medium was changed every 2 or 3 days.

### 2.5. Online TEER Measurement with Staurosporine Exposure

Caco-2 cells were cultured using the aforementioned cell culture method, and drug exposure tests were conducted on days 13–21 of the culture when the TEER values remained relatively stable. The drug exposure test involved the following steps: First, 650 μL of DMEM containing 0.1 μM or 1.0 μM staurosporine (STA: S4400, Sigma-Aldrich, St. Louis, MO, USA) was added into both the inlet and outlet of the medium reservoirs of the top channel with a multipipette. Subsequently, 650 μL of STA-free DMEM was added into the inlet and outlet of medium reservoirs with a multipipette on the bottom channel. After filling the medium in the reservoirs, the tubes were filled with the same concentration of STA-containing DMEM for the top and STA-free DMEM for the bottom. Perfusion culture was performed using a peristaltic pump. During the drug exposure study, the TEER measurement station equipped with the Fluid3D-X TEER and perfusion setup was placed in a CO_2_ incubator. TEER measurements were performed at 3 min intervals until 16 h after the onset of the measurements. Lucifer yellow (LY) permeability assays were performed 20 h after STA exposure. Subsequently, cells were fixed in 4% paraformaldehyde for immunostaining.

### 2.6. Lucifer Yellow Permeation Assay

Before the permeability assay, the medium in the channels, as well as the inlet and outlet reservoirs, were replaced with a transport buffer (TP buffer, HBSS with Ca^2+^ and Mg^2+^, containing 10 mM HEPES, pH 7.4, phenol-red-free, FUJIFILM Wako Pure Chemical Corporation, Osaka, Japan). The chips were incubated under 37 °C 5% CO_2_ for 30–60 min. Following acclimation, the TP buffer in both the inlet and outlet reservoirs was removed using a multipipette. Subsequently, 500 µL of 100 µM Lucifer yellow (LY, 128-06271, FUJIFILM Wako Pure Chemical Corporation, Osaka, Japan) in the TP buffer was applied to the inlet reservoir of the top channel. To ensure an even distribution of LY in the channel, 200 µL of the LY solution was collected from the outlet reservoir and returned to the inlet twice.

The same process was repeated with 500 µL of the TP buffer for the bottom channel. Subsequently, 100 µL of the solution was collected from both the top and bottom outlets to serve as samples at 0 h. The chips were then incubated at 37 °C with 5% CO_2_. After 30 min, the same procedure was repeated, and 100 µL of the solution collected from the top and bottom outlets served as a sample at 0.5 h.

Samples collected at each time point were analyzed using a microplate reader (SH-9500Lab, CORONA ELECTRIC, Ibaraki, Japan) at Ex/Em: 485 nm/538 nm. The transmission coefficient was calculated using Equation (2) [21,22].

(2) $$P_{app}=\frac{dQ}{dt}\cdot \frac{1}{C_{0}\cdot S}$$

Here, *Papp* (cm/s) represents the transmission coefficient, *dQ*/*dt* (nmol/s) represents the transmission velocity, *C_0_* represents the initial concentration of LY on the apical side, and *S* represents the surface area (0.65 cm^2^) of the porous membrane.

### 2.7. Immunocytochemistry

To detect ZO-1 and phalloidin protein expression in Caco-2 cells, the cells were cultured in Fluid3D-X TEER and fixed using 4% paraformaldehyde. Following blocking with 1% bovine serum albumin in PBS, the cells were incubated overnight with rabbit anti-ZO-1 antibody (Proteintech Group, Rosemont, IL, USA). Next, the cells were incubated with a secondary antibody, Alexa Fluor 568 donkey anti-rabbit IgG (Invitrogen, Carlsbad, CA, USA) for 1 h. Subsequently, Acti-Stain 488 Fluorescent Phalloidin (Cytoskelton, Denver, CO, USA) and DAPI solution (DOJINDO LABORATORIES, Kumamoto, Japan) were added. Finally, the cells were mounted in a VECTASHIELD medium (Vector Laboratories, Burlingame, CA, USA).

Cell imaging was performed using a confocal microscope (A1R; Nikon, Tokyo, Japan) at the Tokai University Imaging Center for Advanced Research.

### 2.8. Statistical Analysis

All values are expressed as mean ± S.D. from at least three independent experiments. Tukey’s *t*-test was performed for paired and unpaired comparisons, as appropriate, and differences were considered significant at *p* < 0.05.

## 3. Results and Discussion

### 3.1. Evaluation of Electrode Design Through FEM Simulations

To determine the optimal Au electrode design for precise TEER measurements, the uniformity of current density in two different electrode designs was assessed using FEM analysis (Figure 2a,b). The *ΔI* value was lower in Model 2 compared with that in Model 1, with Model 2 demonstrating a more consistent current density distribution (Figure 2c). The observed outcome may be attributed to the configuration of Model 2, where the distance between the upper and lower electrodes was maximized, resulting in a lower likelihood of current flow. Conversely, the proximity between the electrodes was minimized, leading to a higher probability of current flow. As previously mentioned, electrodes should be positioned across the entire surface of the target being measured. Ideally, the ratio between the longest and shortest distances over which current flows (longest distance/shortest distance) should be as close to 1 as feasible. This ensures a more uniform current density distribution [15]. In this experiment, Model 1 had the longest/shortest distance ratio of 1.20, whereas Model 2 had a ratio of 1.05. Given that the distance ratio in Model 2 was closer to 1, a more uniform current density distribution was anticipated. Based on the findings from the aforementioned investigations, the Fluid3D-X TEER was fabricated using the electrode design from Model 2 in this study.

A comparison between the double-layer channel-type MPS chip—Fluid3D-X, made of PET and used in this study, and the Emulate chip, made of PDMS—revealed that midazolam adsorption, not observed in Fluid3D-X, was detected in the latter. This indicates that drug adsorption occurred due to the PDMS material [20]. Because the chip created in this study was also made of PET, a significant reduction in drug adsorption and sorption was anticipated compared with that of PDMS. Hence, it is considered a useful tool for drug development because it maintains a relatively constant drug concentration within the flow channel during drug testing.

### 3.2. TEER Measurement for Caco-2 Perfusion Culture

**Figure 3:**
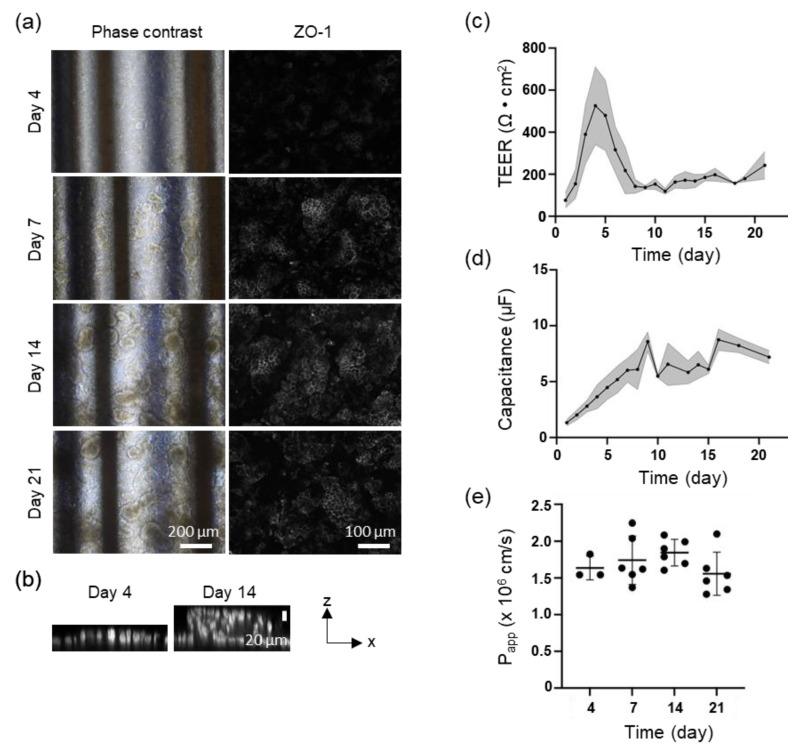
Effects of perfusion culture of Caco-2 cells on the Fluid3D-X TEER on TEER, capacitance, and Lucifer yellow permeability (*Papp*). Caco-2 cells were cultured with medium perfusion (the perfusion rate is described in Section 2.4). (**a**) Representative phase contrast and immunohistostaining of Caco-2 cells at 4, 7, 14, and 21 days after seeding; magnification ×100 (phase contrast) and ×200 (ZO-1 immunohistostaining). (**b**) Representative layer thicknesses of Caco-2 cells. Blue: DAPI, red: ZO-1; magnification ×200. (**c**) Time course of the actual TEER value of Caco-2 cells on Fluid3D-X TEER, n = 23 (eight independent experiments, triplicate or quadruplicate chips/experiment excluding chips with electrode defects or problems with cell seeding). Mean and error with standard deviation, gray area means the area within error bands. (**d**) Time course of the actual capacitance value of Caco-2 cells on Fluid3D-X TEER, n = 17 (six independent experiments, triplicate or quadruplicate chips/experiment, excluding chips with electrode defects or problems with cell seeding). Mean and error with standard deviation, gray area means the area within error bands. (**e**) LY permeation test results. The Papp results are obtained at each time point on day 4 (n = 1, one experiment, triplicate chips per experiment), as well as on days 7, 14, and 21 (n = 2, two independent experiments, triplicate chips per experiment). Each dot represents the actual value of *Papp*.

Perfusion culture on Fluid3D-X TEER revealed significant findings regarding the development of Caco-2 cells. By day 4, the cells demonstrated nearly monolayer-like structures, which progressed to villi-like structures by day 7. These villi-like structures continued to mature over time (Figure 3a,b) [21]. The expression of ZO-1, a tight junction protein, was first observed on day 4, particularly on the apical side of the villi-like structures as they became more developed (Figure 3a). The TEER value peaked at 500–600 Ω·cm^2^ between days 4 and 5 when the cell morphology was monolayer-like and then decreased as the villi-like structures matured, stabilizing at approximately 150–200 Ω·cm^2^ (Figure 3c). The capacitance increased progressively, reaching a peak on day 9 and remaining around 6 µF from day 10 to day 15, before saturating around 8–9 µF after a further increase on day 17. Permeability was assessed using LY at various time points, with no significant differences observed on days 4, 7, 14, and 21 (Figure 3e).

As shown in Figure 3c, the TEER values decreased as the morphology of the Caco-2 cells transitioned from a monolayer to a villi-like structure. As reported in a previous study, TEER decreases when Caco-2 cells form villi-like structures in perfusion culture [11]. TEER reflects the ion permeability of the intercellular space. As the tight junction becomes tighter, the TEER value increases. In the small intestinal mucosa, intercellular spaces contribute over 90% to the total membrane resistance. The TEER value serves as an indicator of ion transport through intercellular spaces, which is a pathway with low resistance [23]. The TEER value of the small intestine is approximately 50–100 Ω·cm^2^ [6]. The TEER value of the tissue may closely resemble that of the small intestine owing to the villi-like structure formed by the perfusion culture of Caco-2 cells, mimicking the function of the small intestine wall in vivo. This is further supported by the consistent LY *Papp* values despite a decrease in TEER values after day 7, when Caco-2 cells began to form villi-like structures.

In this study, capacitance increased over time. The capacitance of the plasma membranes is approximately 0.5–1 μF [24]. In contrast, studies conducted using cardiomyocytes have revealed a direct relationship between cell volume and capacitance, indicating that capacitance increases as cell volume increases [25]. Additionally, capacitance is recognized as a reflection of the cell surface area [26]. Nikulin et al. [27] found that capacitance continued to rise even after TEER reached a plateau in Caco-2 cells on an insert, suggesting that this increase resulted from an expansion in the cell surface area. They observed that capacitance increased post-TEER plateau, indicating cell maturation, specifically microvilli formation in Caco-2 cells. In undifferentiated cells, the surface areas of the apical and basal sides are nearly identical. However, as differentiation increases, the basal surface area remains constant, while the apical surface area increases due to the formation of microvilli. Consequently, capacitance reaches approximately twice that of the undifferentiated state [27]. Similarly, MPS studies have reported increases in capacitance with microvilli formation, corresponding to an increased surface area [28]. In addition, a previous study using Fluid3D-X and Caco-2 cells demonstrated that as villi-like structures developed, both the ratio of relative object area, which indicates the area of villi-like structures, and sum intensity, which indicates the density of villi-like structures, increased by employing NIS-Elements software ver. 6.01.00 (Nikon Corporation, Tokyo, Japan) [21]. Therefore, the increase in capacitance over time in the present study indicates an increase in the surface area and volume owing to the transformation of Caco-2 cells into villi-like structures. The increase in capacitance post-transformation reflects the development of microvilli as cells reach a high level of differentiation. Changes in capacitance serve as a valuable indicator of morphological alterations in cells [29], such as the formation of microvilli and villi-like structures. Therefore, capacitance can be crucial in understanding and identifying changes in cell morphology.

### 3.3. Online TEER Measurement with Drug Exposure

**Figure 4:**
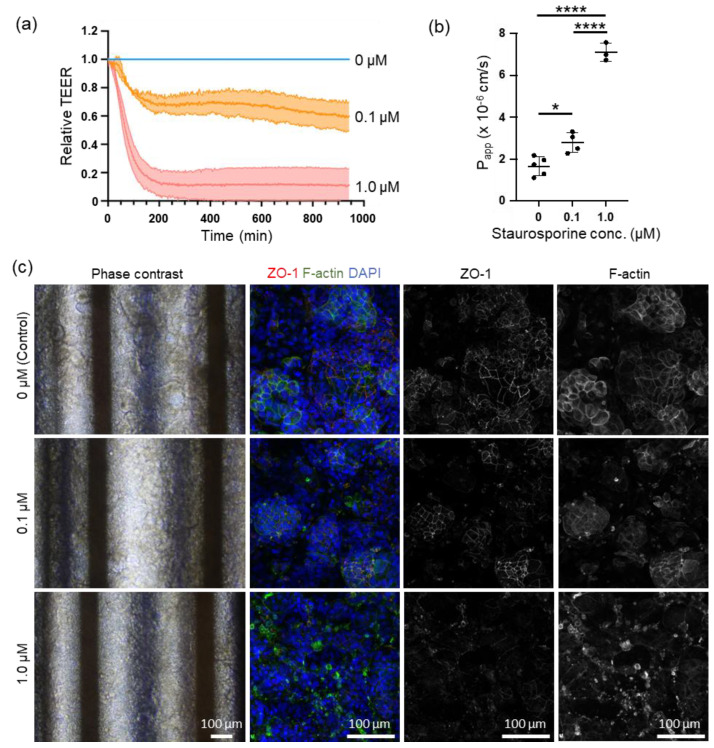
Effect of staurosporine (STA) on Caco-2 cells on real-time TEER and capacitance monitoring, and Lucifer yellow permeability assay (*Papp*). Caco-2 cells were cultured using the medium perfusion described in Section 2.4. (**a**) Real-time monitoring of the relative value of TEER. The relative TEER value was normalized twice, with the TEER value at time zero and control. Blue: control (n = 4, four independent experiments); orange: 0.1 μM STA exposure (n = 3, three independent experiments); red: 1.0 μM STA exposure (n = 3, three independent experiments). (**b**) Lucifer yellow permeability (*Papp*) results obtained from Caco-2 cells exposed to each concentration of STA, n = 5 (control), n = 4 (0.1 μM STA), and n = 3 (1 μM STA). n = 4 (four independent experiments, triplicate or quadruplicate chips/experiment excluding chips with electrode defects or problems with cell seeding.) (**c**) Representative phase contrast and immunohistostaining of Caco-2 cells on the 16–18th days after seeding; magnification ×40 (phase contrast) and ×200 (ZO-1 and phalloidin immunohistostaining), scale bar 100 μm; values were expressed as mean ± standard deviation; * *p* < 0.05, **** *p* < 0.0001.

We conducted online measurements of cell motility using STA to investigate the feasibility of this system for testing drug efficacy and toxicity. In a study examining epithelial barrier damage caused by STA exposure, an online measurement of TEER revealed a decrease in relative TEER compared with the control group over time following exposure to 0.1 µM and 1.0 µM of STA, peaking at approximately 4 h post-exposure. The relative TEER values dropped to 0.6–0.8 and 0–0.2 for the 0.1 µM and 1.0 µM STA exposures, respectively, before stabilizing (Figure 4a). Capacitance measurements were performed before and after STA exposure, with a noticeable decline observed at 0.1 µM of STA and an inability to measure capacitance at 1.0 µM of STA (Supplementary Figure S1a). Following exposure to 1.0 µM STA, the capacitance was monitored online for up to 200 min at 3 min intervals, showing a gradual decrease post-exposure (Supplementary Figure S1b). The LY permeability assay conducted after STA exposure demonstrated a significant dose-dependent increase in *Papp* (Figure 4b). Additionally, changes in cell morphology were observed following STA exposure, with alterations evident at varying concentrations of STA utilized (Figure 4c). Phase-contrast images revealed a reduction in villi-like structures seen in the control group with 0.1 µM of STA, whereas these structures collapsed with 1.0 µM of STA. Immunocytochemistry analysis revealed that both ZO-1 and F-actin, which bind to ZO-1, were present in the intercellular spaces in the control. However, exposure to 0.1 µM STA resulted in a decrease in the expression intensity of ZO-1 at the intercellular spaces and a shift in the distribution of F-actin from the intercellular spaces to the cytoplasm. Exposure to 1.0 µM STA resulted in the collapse of ZO-1 expression at intercellular spaces and aggregation of F-actin.

STA, an indolocarbazole antibiotic discovered in Japan by Omura et al., possesses antibacterial, antifungal, antihypertensive [30,31], and antiplatelet aggregation effects [32]. Additionally, STA inhibits the activity of protein kinase C by preventing ATP from binding to kinase [33], which activates caspase-3 and induces apoptosis by arresting cells in the G1/G2 phase of the cell cycle [34,35]. Recently, clinical trials on hematological and solid tumors have focused on their anticancer activities [36]. In contrast, STA induces epithelial barrier disruption, and its exposure increases FITC-dextran leakage [37] and decreases TEER [38].

The findings of this experiment demonstrated a dose-dependent decrease in TEER induced by STA. Tight junction injury, caused by drugs or inflammatory cytokines, such as Tumor Necrosis Factor-alpha, resulted in a reduction in the expression level of ZO-1. ZO-1 served as an anchor for tight junction proteins, facilitating their binding to intracellular actin filaments. This decrease in ZO-1 expression caused the relaxation of tight junctions and increased permeability of the intercellular space [7], ultimately leading to a decrease in TEER.

Observations from phase-contrast microscopy and immunostaining revealed that treatment with 0.1 µM STA maintained the villi-like structures of Caco-2 cells and considerably preserved tight junctions. Additionally, cellular damage caused by apoptosis was mild, resulting in a 30% decrease in TEER compared with control cells. However, treatment with 1.0 μM STA induced significant damage to tight junctions and widespread apoptosis, leading to a substantial decrease in TEER across a broad range of cells.

In a study on cell viability and capacitance induced by doxorubicin, Lee et al. reported that apoptosis decreased capacitance [39]. Furthermore, Park et al. reported that doxorubicin reduced capacitance in breast cancer cells in a dose-dependent manner, and this reduction correlated with the WST assay results within the 0–2μM range [40]. In intestinal epithelial cells, the loss of blebbing and microvilli during apoptosis results in a reduced capacitance [41]. A similar outcome was observed in this study at 1.0 µM STA exposure, which induced apoptosis. Real-time capacitance monitoring can therefore detect cell death earlier and be applicable for the real-time monitoring of cell viability, highlighting the effectiveness of this approach [42]. Moreover, recent research has revealed that proximal tubular cells cultured in a double-layer microfluidic chip equipped with a TEER measurement device also demonstrated cisplatin-induced damage. This damage impacts both TEER and capacitance in monolayer culture systems. Additionally, an increase in capacitance was observed as a result of cell detachment from the extracellular matrix owing to epithelial barrier dysfunction [9]. In our study, the cultured intestinal epithelial cells demonstrated villi-like structures, indicating that STA exposure may have disrupted these structures, resulting in a decrease in surface area compared with monolayer culture systems.

In conclusion, our findings suggest that this system is suitable for evaluating cell kinetics in drug efficacy and toxicity studies, as it can measure cell kinetics in detail by measuring TEER and capacitance online.

## 4. Conclusions

In this research, we developed a double-layer microfluidic chip with an integrated TEER measurement function and thoroughly assessed its functionality. Conventional double-layer microfluidic chips have several limitations, including light diffusion through the porous membrane, which hinders cell observation, inconsistent current density during TEER measurements, and the drug adsorption and sorption properties of PDMS, which affect drug testing accuracy. To address these limitations, we designed an electrode pattern that ensured optimized current density uniformity through FEM simulations. Additionally, we developed double-layer microfluidic chips made of PET material and equipped them with these electrodes. Although several double-layer channel-type MPS chips with TEER measurement functions have been reported [16], the Fluid3D-X TEER developed in this study, by utilizing PET as its material, overcomes issues related to drug adsorption and sorption. In addition, it enables uniform TEER measurement across the cell culture area, making it an unprecedentedly useful tool. To assess the performance of this system, we conducted perfusion culture experiments with Caco-2 cells to monitor changes in TEER and capacitance over time. Our findings reveal a correlation between the transformation of Caco-2 cells from a monolayer structure to a three-dimensional villi-like structure over time and changes in TEER and capacitance. Furthermore, we conducted online measurements of cell kinetics using STA to evaluate the system’s suitability for drug efficacy and toxicity studies. The TEER measurements validated that STA negatively impacts intestinal epithelial barrier function in a dose-dependent manner. Additionally, a decrease in capacitance was observed following STA exposure, suggesting a link to the induction of apoptosis. Overall, our results suggest that this system offers the real-time assessment of cell barrier function and can enhance the accuracy of drug efficacy and toxicity testing in the drug discovery process.

The results of this study will significantly enhance TEER measurement technology in microfluidic chips, particularly resulting from the utilization of PET material, which minimizes drug adsorption. This advancement will significantly improve the reliability of drug testing. Indeed, the experimental data demonstrated high reproducibility, and a mass production process via stacking has been established with the goal of commercializing our chips. Moreover, this innovative system is not limited to just intestinal models but can also be applied to various organ models, such as the blood–brain barrier and renal tubules. Future studies are expected to further develop this technology and enable a more comprehensive analysis of cellular responses by integrating TEER measurements with other cellular assessment methods. This will ultimately position this method as a valuable alternative to animal testing in the drug discovery process. Regarding drug response assessment, this study also demonstrated high reproducibility, and we believe our chips will serve as a useful tool for evaluating drug toxicity and efficacy. Therefore, we are confident that this system is not merely in a preliminary stage but has reached a practical stage. The results of this study will help advance MPS technology and represent a significant stride toward enhancing the precision of drug discovery and toxicity testing.

One limitation of this study is the utilization of Au as the electrode material, which hinders the observation of brightfield images and results in low-throughput measurements. This issue can be addressed by utilizing transparent ITO as the electrode material, a solution that is also applicable when transitioning to ITO from another material. Improving the measurement station to increase throughput is a viable option, although it poses a trade-off between throughput and space constraints owing to the technical hurdles involved. Nonetheless, our proposed system promises to significantly expand the scope of MPS applications, potentially offering an alternative to animal experiments. Our future research will focus on exploring the practical implications of this system in the drug discovery process.

## Data Availability

The authors confirm that the data supporting the findings of this study are available within the article [and/or] its Supplementary Materials.

## Supplementary Materials

The following supporting information can be downloaded at: https://www.mdpi.com/article/10.3390/bios15100663/s1, Figure S1: Variation in capacitance in the STA exposure study; Table S1: Parameters utilized in the FEM simulation; Table S2: Boundary conditions utilized in the FEM simulation; Table S3: Specifications of the system.
