# Supplementary material for: Double-Layered Microphysiological System Made of Polyethylene Terephthalate with Trans-Epithelial Electrical Resistance Measurement Function for Uniform Detection Sensitivity
Source: Biosensors (Basel). 2025 Oct 2;15(10):663. doi: 10.3390/bios15100663 (PMC12562628; doi:10.3390/bios15100663)
Supplement: Supplementary file 1 [file biosensors-15-00663-s001.zip › biosensors-3857065-supplementary.pdf]

Supplementary Information for

Double-layered microphysiological system made of polyethylene terephthalate with trans-epithelial electrical resistance measurement function for uniform detection sensitivity

Naokata Kutsuzawa [1,2,3], Hiroko Nakamura [1], Laner Chen [1], Ryota Fujioka [4], Shuntaro Mori [4], Noriyuki Nakatani [4], Takahiro Yoshioka [5] and Hiroshi Kimura [1,*]

1. Micro/Nano Technology Center, Tokai University, Hiratsuka, Kanagawa, 259-1292, Japan
2. Division of Pulmonary Medicine, Department of Medicine, Tokai University School of Medicine, Isehara, Kanagawa, 259-1143, Japan
3. The Institute of Medical Sciences, Tokai University School of Medicine, Isehara, Kanagawa, 259-1143, Japan
4. SCREEN Holdings Co., Ltd., Kyoto, Kyoto, 612-8486, Japan
5. TOKYO OHKA KOGYO Co., Ltd., Koza, Kanagawa, 253-0114 Japan

* Correspondence: author: hkimura@tokai.ac.jp

This PDF file includes:

Figure S1

Tables S1 to S3

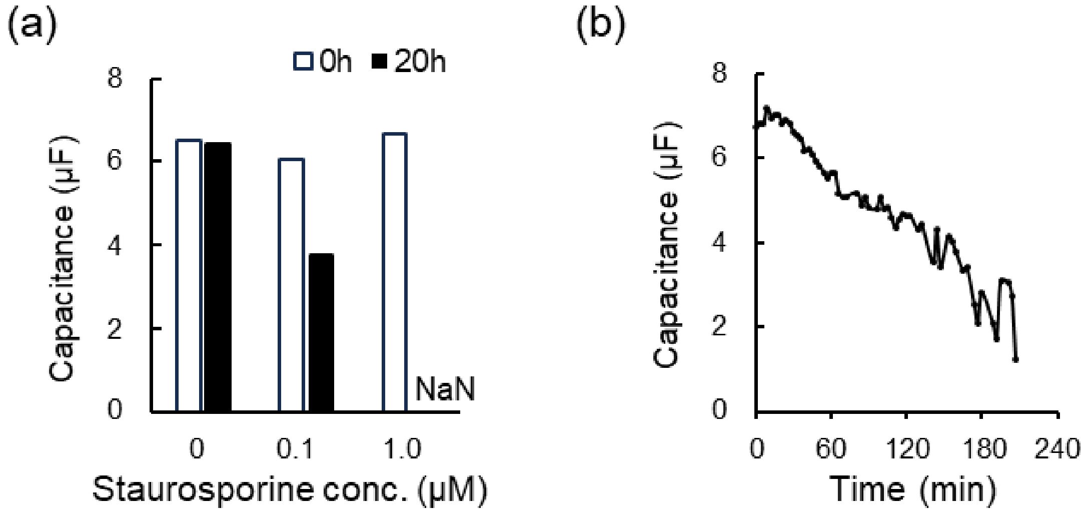

Figure S1. Variation in capacitance in the STA exposure study. (a) Capacitance change with STA concentration. Left bar: actual capacitance value at 0 h of exposure, right bar: actual capacitance value at 20 h after exposure. (b) Real-time monitoring of capacitance after 1.0 μM STA exposure.

Table S1. Parameters utilized in the FEM simulation

Parameters	
Width of cell culture area	2,000 μm
Length of cell culture area	3,400 μm
Height of cell culture area	Top channel: 710 μm<br>Bottom channel: 710 μm
Porous membrane thickness	12 μm
Au electrode thickness	0.1 μm
Membrane conductivity	1.0×10^{-2} S/m
Conductivity of the medium	1.5 S/m
Electrode conductivity	1.4×10^{4} S/m
Input voltage	10 mV

Table S2. Boundary conditions utilized in the FEM simulation

Boundary conditions	
Working electrode (Top)	10 mV
Working electrode (Bottom)	Ground
Reference electrode	Floating potential
Other part	Insulation

Table S3. Specifications of the system

Measurement range (accuracy)	100 ~ 30,000 Ω (±3%)
Resolution	1 Ω
TEER measurement condition	Current 1 μA<br>Frequency 11.5 Hz (sine wave)
Impedance measurement condition	Current 1 μA<br>Frequency 10 Hz–100 kHz (sine wave)
Operating environment	Room temperature
